# Secukinumab mitigates diethyl nitrosamine-induced acute liver injury in mice via modulating p-ERK/p-eIF2α/CHOP and NLRP3/Caspase-1/IL-1β pathways

**DOI:** 10.1007/s00210-026-05142-x

**Published:** 2026-03-27

**Authors:** Zaid F. AL-rasheedi, Omnia A. Nour, Dalia H. El-Kashef

**Affiliations:** Department of Pharmacology and Toxicology, Faculty of Pharmacy, Mansoura University, Mansoura, 35516 Egypt

**Keywords:** Secukinumab, Diethyl nitrosamine, Mice, p-ERK/p-eIF2α/CHOP, NLRP3/Caspase-1/IL-1β

## Abstract

Secukinumab is a fully humanized monoclonal antibody which selectively targets IL-17A. It showed anti-inflammatory, neuroprotective, and antioxidant activity in many experimental studies. This study was aimed at exploring the hepatoprotective potential of secukinumab against diethylnitrosamine (DEN)-induced acute liver injury in mice, with an emphasis on its anti-inflammatory, antioxidant, and antipyroptotic properties. Thirty-six male mice were randomly divided into six groups—group one (Control): mice did not receive any treatment; group two (SEC20): mice received SEC (20 mg/kg, i.p.) only; group three (DEN group): mice received DEN (150 mg/kg, i.p.); group four (SEC5 + DEN): mice received SEC (5 mg/kg, i.p.) then injected with DEN 1 h after SEC administration; group five (SEC10 + DEN): mice received SEC (10 mg/kg, i.p.), then injected with DEN 1 h after SEC administration; and group six (SEC20 + DEN): mice received SEC (20 mg/kg, i.p.) then injected with DEN 1 h after SEC administration. Compared to DEN-injected mice, secukinumab at the dose of 5, 10, and 20 mg/kg significantly decreased serum levels of ALT, AST, GGT, and LDH as well as hepatic levels of MDA, IL-17A, p-ERK, p-eIF2α, CHOP, NLRP3, and IL-1β, in addition to increased serum level of albumin as well as hepatic levels of GSH and TAC. Histopathologically, secukinumab attenuated DEN-induced histopathological irregularities in hepatic tissues. Moreover, secukinumab markedly reduced the hepatic expression of NF-κB and Caspase-1. Secukinumab at the doses (5, 10, and 20 mg) showed hepatoprotective, antioxidant, and anti-inflammatory effects against DEN-induced hepatotoxicity in mice.

## Introduction

Although the liver is the primary organ involved in detoxification, exposure to certain medications, bacterial or viral infections, environmental xenobiotics, and anticancer treatments can all injure the liver and induce a variety of liver disorders. Due to its critical function in metabolism and its capacity for xenobiotic concentration and biotransformation, the liver is more vulnerable to damage than any other organ (El-Kashef and Sharawy 2023). It is commonly known that the majority of nitrosamines are strong carcinogens that can cause tumors in a number of human organs, including the liver, kidney, brain, stomach, and esophagus. Humans are exposed to diethylnitrosamine (DEN) and other nitrosamines due to the consumption of large quantities of nitrite-cured meat, processed and fried foods, and cigarettes on a regular basis (Shaker et al. 2022). DEN mainly affects the liver, where it undergoes biotransformation through cytochrome P450-dependent processes that are most active in centrilobular hepatocytes (Kang et al. 2007).

Endoplasmic reticulum (ER) stress has a number of harmful effects on cells and tissues. Crucially, ER stress causes the unfolded protein response (UPR) to be stimulated, which reduces the load of unfolded proteins and increases the ability of proteins to fold to preserve protein homeostasis. Phosphorylated extracellular signal regulated kinase (p-ERK), a typical route for the inflammatory response brought on by ER stress, is involved in UPR activation and may increase the production of the proapoptotic proteins comprising C/EBP homologous protein (CHOP) in advanced stages. Most significantly, ER stress may result in the production of a significant amount of reactive oxygen species (ROS) (El-Waseif et al. 2025).

Pyroptosis is an inflammatory programmed cell death that contributes to liver dysfunction and is distinguished by activated pyrin domain containing 3 (NLRP3) of the NLR family. The multiprotein complex known as the NLRP3 inflammasome is crucial for a number of illnesses, including inflammatory conditions. One of the potent inducers of NLRP3 inflammasome activation is oxidative stress. Caspase-1 protein activation and subsequent interleukin-1 beta (IL-1β) activation and secretion were catalyzed by NLRP3 (Abo El-Magd et al. 2023).

Interleukin-17A, a proinflammatory cytokine, is released by NK cells, CD8-positive T cells, helper T cells (Th17), and neutrophils. IL-17A causes release of cytokines and chemokines, which attract neutrophils and monocytes to the site of inflammation. It has been demonstrated that IL-17A is crucial for a number of illnesses including multiple sclerosis, inflammatory bowel disease, rheumatoid arthritis (Sun et al. 2016), and DEN-induced liver injury (Shaker et al. 2022).

Secukinumab is a fully human monoclonal antibody that selectively targets IL-17A. It has been approved to treat a variety of inflammatory conditions, such as moderate to severe psoriasis and psoriatic arthritis. Also, it has been reported that secukinumab exhibited neuroprotective and antioxidant properties in an experimental model of multiple sclerosis (Abdel-Maged et al. 2020). Another study revealed that secukinumab-induced selective suppression of IL17A signaling reduced proinflammatory molecules in human astrocytes (Elain et al. 2014).

This study was carried out to explore the hepatoprotective potential of secukinumab in a mouse model of diethylnitrosamine (DEN)-induced acute liver injury, with an emphasis on its anti-inflammatory, antioxidant, and antipyroptotic properties.

## Materials and methods

### Animals

Male BALB/c albino mice weighing 25–30 g were supplied with food pellets and water throughout the acclimatization and experimental periods. Mice were placed in plastic cages at a temperature of 25 °C, allowed free food and water and sustained on a 12-h light/dark cycle. The care and methods used for the experimental animals met the standards set by the National Institutes of Health and the guidelines approved by the Mansoura University Animal Care and Use Committee (MU-ACUC) (code number PHARM.MS.24.05.30).

### Drug and chemicals

Secukinumab (SEC) was supplied as a pharmaceutical preparation (COSENTYX ®) pre-filled pen (150 mg/1 ml). Diethylnitrosamine (DEN) was supplied as a liquid vial containing 99%/10 ml (Sigma, USA; density 0.95 g/mL; Cat. no. 73861). Both SEC and DEN were diluted in sterile normal saline and were administered intraperitoneally.

### Experimental design

There were six groups of six mice in each:

- The first group (CTR): mice did not receive any treatment.
- The second group (SEC): Mice received SEC (20 mg/kg, i.p.) only (Grigsby et al. 2020).
- The third group (DEN group): Mice received DEN (150 mg/kg, i.p.) (Shaker et al. 2022).
- The fourth group (SEC5 + DEN): Mice received SEC (5 mg/kg, i.p.) (Grigsby et al. 2020) then injected with DEN 1 h after SEC administration.
- The fifth group (SEC10 + DEN): Mice received SEC (10 mg/kg, i.p.)(Karatas et al. 2021), then injected with DEN 1 h after SEC administration.
- The sixth group (SEC20 + DEN): Mice received SEC (20 mg/kg, i.p.) (Grigsby et al. 2020) then injected with DEN 1 h after SEC administration.

**Fig. 1:**
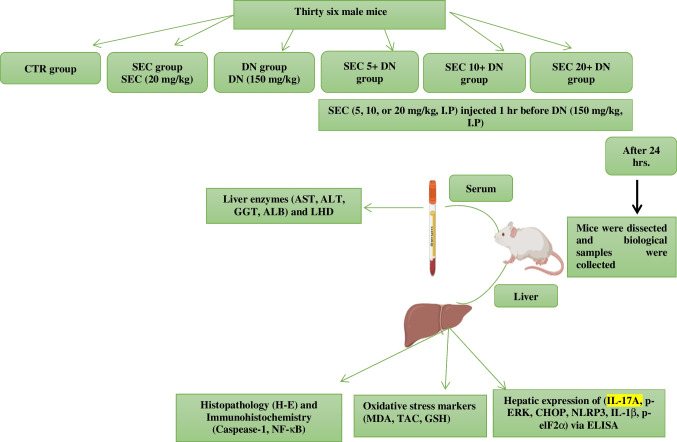
schematic diagram for experimental protocol

Twenty-four hours after DEN injection, mice were anesthetized by secobarbital (Sigma Pharmaceuticals, Egypt) with dose (50 mg/kg, i.p.), and blood samples were obtained. After that, serum was separated by centrifuging them at 4000 g for 10 min. Additionally, liver tissue was isolated and divided into 2 portions; one portion was stored at − 80 °C for further analysis and the other was kept and fixed by immersing them in 10% (v/v) buffered formalin for histopathology and immunohistochemistry procedures. The above mentioned protocol is illustrated in Fig. 1.

### Determination of liver biomarkers

The extent of liver injury in mice was evaluated by measuring alanine aminotransferase (ALT; cat. no. 11409005), aspartate aminotransferase (AST; cat. no. 11408005), gamma-glutamyl transferase (GGT; cat. no. 11416001), lactate dehydrogenase (LDH; cat. no. LDH25), and albumin (ALB; cat. no. 211011) levels in serum samples by kinetic kits (LiquiCHECK, Spectrum-diagnostics, Egypt).

### Determination of oxidative stress

Levels of reduced glutathione (GSH), a measure of nonenzymatic antioxidants, total antioxidant capacity (TAC), and contents of malondialdehyde (MDA), a lipid peroxidation marker, were assessed in the liver homogenates using commercial kits from Biodiagnostic (Egypt), in accordance with the instructions given.

### Enzyme-linked immunosorbent assay (ELISA)

Using ELISA kits, mouse C/EBP Homologous Protein (CHOP; Cat. no. EM1933, FineTest), mouse NLR Family, Pyrin Domain Containing Protein 3 (NLRP3; Cat. no. orb782216, Biorybt), mouse phosphoeukaryotic translation initiation factor 2 subunit (p-eIF2α; Cat. No. ABIN1380564, Antibodies-online), mouse interleukin 1 Beta (IL-1β; Cat. no. EM0109, Finetest), mouse interleukin 17 A (IL-17A; Cat. no. E-EL-M0047, Elabscience), and mouse phosphoextracellular signal-regulated kinase (p-ERK; Cat. no. MBS250034, MyBiosource) were measured in hepatic tissue homogenates. A protease inhibitor (Roche, Germany; Cat. no. 11836170001) was added to 0.9 ml of cooled lysis buffer (150 mM NaCl, 0.5% v/v Triton X-100, 10 mM Tris pH 7.4) after 0.1 g of liver had been minced. The tubes were then centrifuged at 5000 g for 10 min at 4 °C. The manufacturer’s instructions were followed for loading the separated supernatants of liver lysis onto precoated ELISA plates with the proper capture antibody after they had been prediluted with phosphate buffered saline in a 2:1 ratio. The lysed liver samples' protein content was also calculated (Bradford 1976).

### Histopathology

Graded concentrations of ethanol in water (70, 80, 95, and 100% v/v) were used to first dehydrate the fixed liver tissues in the plastic cassettes. They were then submerged in an ethanol:xylene (1:1) solution and, lastly, pure xylene solution to eliminate any remaining residues of ethanol. Liver tissues were then embedded in paraffin blocks and sliced into 4 mm thick sections using a microtome, which were then transferred onto glass slides. Hematoxylin and eosin (H&E) staining was then applied after the sections were dewaxed by soaking them twice in xylene solution, then rehydrated in ethanol solutions in the opposite sequence of dehydration (100, 95, and 70% v/v). Finally, the liver sections were examined using a light microscope by the pathologist.

### Immunohistochemistry

Liver slices (paraffin-embedded) were put on coated glass slides for immunohistochemical screening to identify the target proteins being studied. The slides were subsequently put through procedures for non-specific protein binding, endogenous peroxidase elimination, and antigen retrieval. Slide sections were then successively treated with substrate/chromogen, secondary antibodies, and primary antibodies for NF-κBp65 (ab16502) (Abcam; 1:300 dilution) and for Caspase-1 (MA5-16,215) (Thermofisher; 1:500). The Image J program was used to quantify the intensity of staining.

### Statistical analysis

One-way ANOVA was used to examine the parametric data (means ± S.E.M), and the Tukey–Kramer post-test was then performed. All data were statistically analyzed using the GraphPad Prism software (GraphPad Software Inc. V 8.4.2). *P*-values less than 0.05 are regarded as statistically significant.

## Results

### SEC attenuated DEN-induced changes in liver enzymes

**Fig. 2:**
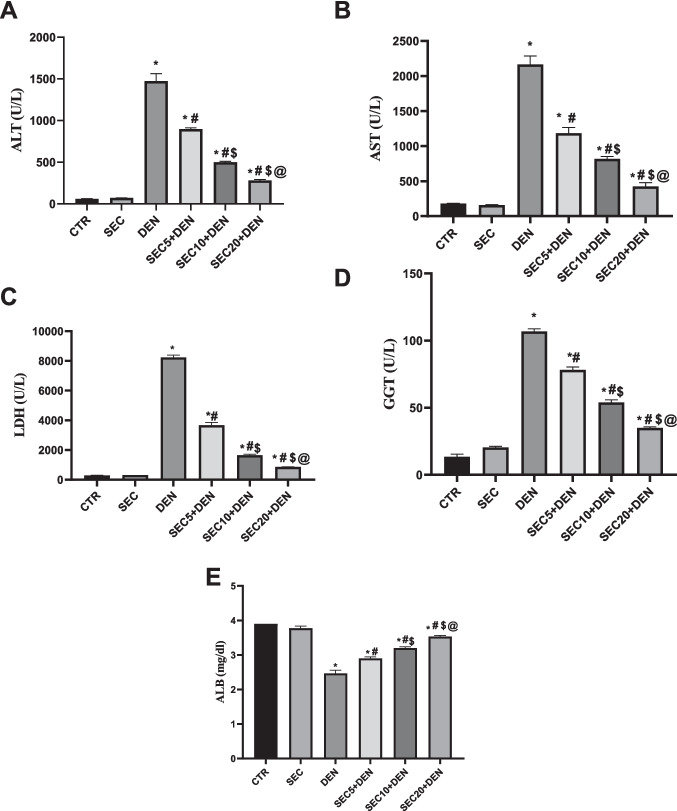
Impact of SEC on DEN-induced changes in liver enzymes. **A** ALT level, **B** AST level, **C** LDH level, **D** GGT level, and (**E**) ALB level. ALB: albumin, ALT: alanine aminotransferase, AST: aspartate aminotransferase, CTR: control, DEN: diethyl nitrosamine, GGT: gamma-glutamyl transferase, LDH: lactate dehydrogenase, and SEC: secukinumab. Data are expressed as means ± S.E.M. (*n* = 6). Mean values were compared via one-way ANOVA followed by post hoc Tukey’s multiple comparison test. *P < 0.05 vs. CTR group, ^#^*P* < 0.05 vs. DEN group, ^$^*P* < 0.05 vs. SEC5 group, and ^@^*P* < 0.05 vs. SEC10 group

Injection of DEN resulted in a significant 24-fold increase in ALT level in comparison to control group (*P* < 0.05, Fig. 2A). Yet, administration of SEC5, SEC10, and SEC20 significantly decreased ALT level by 39%, 66%, and 81%, respectively, compared to DEN group in a dose-dependent effect (*P* < 0.05, Fig. 2A). Concurrently, injection of DEN significantly increased AST level by 12 folds in comparison to control group (*P* < 0.05, Fig. 2B). In a dose-dependent manner, administration of SEC5, SEC10, and SEC20 significantly decreased AST level by 45%, 62%, and 80%, respectively, compared to DEN group (*P* < 0.05, Fig. 2B). Similarly, injection of DEN resulted in a significant 29-fold increase in LDH level in comparison to control group (*P* < 0.05, Fig. 2C). However, administration of SEC5, SEC10, and SEC20 significantly decreased LDH level by 55%, 80%, and 89%, respectively, compared to DEN group in a dose-dependent manner (*P* < 0.05, Fig. 2C).

DEN administration also caused a significant eightfold increase in GGT level in comparison to the control group (*P* < 0.05, Fig. 2D). In a dose-dependent manner, administration of SEC5, SEC10, and SEC20 significantly decreased GGT level by 27%, 49%, and 67%, respectively, compared to the DEN group (*P* < 0.05, Fig. 2D).

Conversely, injection of DEN significantly decreased albumin level by 36.7% in comparison to the control group (*P* < 0.05, Fig. 2E). Administration of SEC5, SEC10, and SEC20 significantly increased albumin level by 1.17, 1.3, and 1.4 fold, respectively, compared to the DEN group in a dose-dependent manner (*P* < 0.05, Fig. 2E).

### SEC attenuated DEN-induced oxidant/antioxidant imbalance

**Fig. 3:**
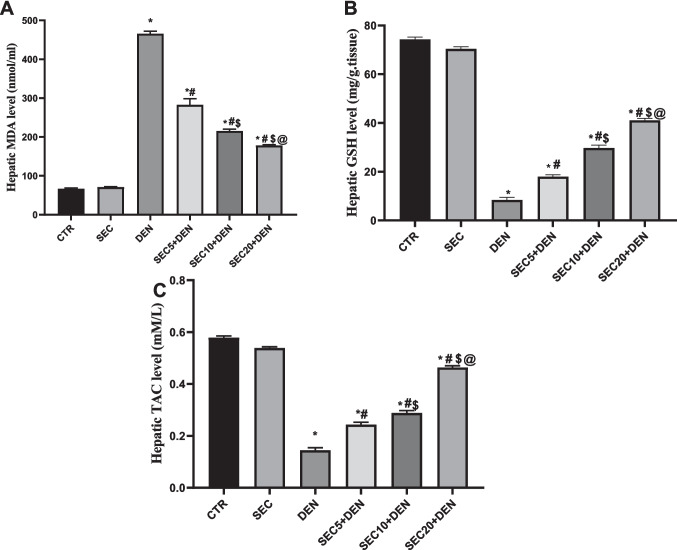
Impact of SEC on DEN-induced changes in oxidant/antioxidant parameters. **A** Hepatic MDA level, **B** hepatic GSH level, and (**C**) hepatic TAC. CTR: control, DEN: diethyl nitrosamine, GSH: reduced glutathione, MDA: malondialdehyde, SEC: secukinumab, and TAC: total antioxidant capacity. Data are expressed as means ± S.E.M. (*n* = 6). Mean values were compared via one-way ANOVA followed by post hoc Tukey’s multiple comparison test. *P < 0.05 vs. CTR group, ^#^*P* < 0.05 vs. DEN group, ^$^*P* < 0.05 vs. SEC5 group, and ^@^*P* < 0.05 vs. SEC10 group

DEN injection induced impairment in oxidant/antioxidant balance as indicated by significant increase in MDA content by 6.5-fold and significant decrease in TAC and GSH levels by 75% and 88%, respectively in comparison to control group (*P* < 0.05, Fig. 3). Administration of SEC5, SEC10, and SEC20 elicited a significant decrease in MDA level by 39%, 53.7%, and 61.7%, respectively compared to DEN group (*P* < 0.05, Fig. 3A). In addition, administration of SEC5, SEC10, and SEC20 resulted in significant 2.14-, 3.5-, and 4.8-fold increases in GSH level in comparison to DEN group (*P* < 0.05, Fig. 3B). Moreover, administration of SEC5, SEC10, and SEC20 resulted in significant 1.7-, 2-, and 3.1-fold increases in TAC in comparison to DEN group (*P* < 0.05, Fig. 3C).

### SEC attenuated DEN-induced changes in IL-17A, p-ERK, CHOP, NLRP3, IL-1β, and p-elF2α levels

**Fig. 4:**
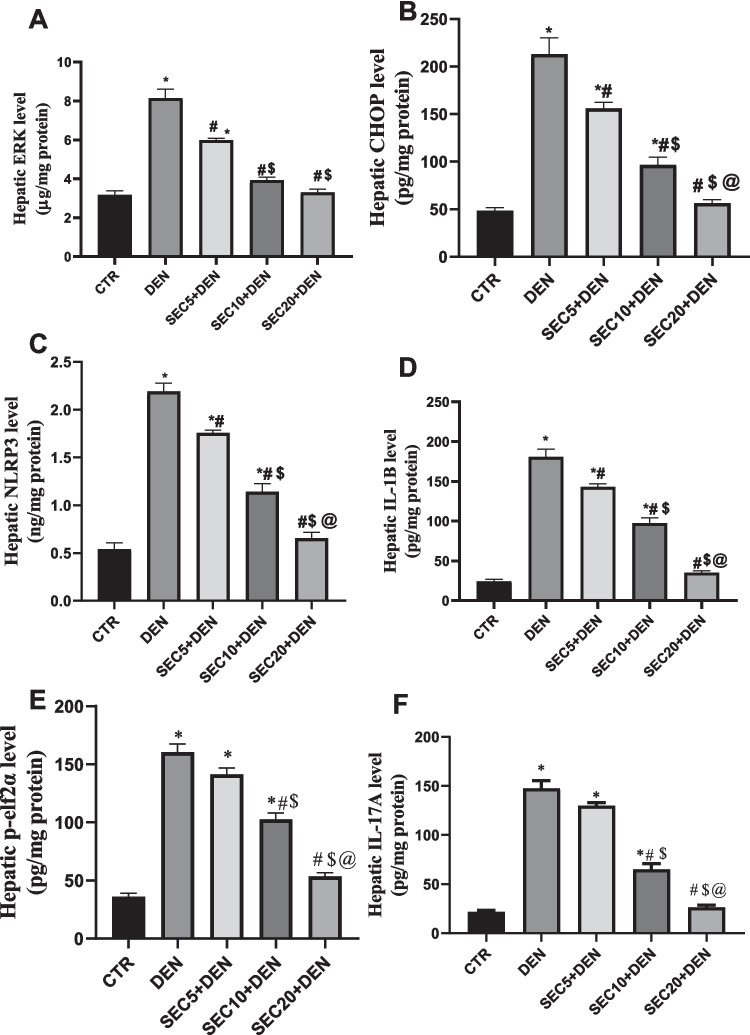
Impact of SEC on DEN-induced changes in hepatic levels of p-ERK**,** CHOP**,** NLRP3**,** IL-1β, p-elF2α, and IL-17A. **A** Hepatic p-ERK level, **B** hepatic CHOP level, **C** hepatic NLRP3 level, **D** hepatic IL-1β level, **E** hepatic p-elF2α level, and **F** hepatic IL-17A level. CTR: control, CHOP: C/EBP homologous protein, DEN: diethyl nitrosamine, p-ERK: phosphoextracellular signal-regulated kinase, IL-1β: interleukin 1-beta, IL-17A: interleukin 17 A, NLRP3: activated pyrin domain containing 3, p-elF2α: phosphoeukaryotic translation initiation factor 2 subunit, and SEC: secukinumab. Data are expressed as means ± S.E.M. (*n* = 6). Mean values were compared via one-way ANOVA followed by post hoc Tukey’s multiple comparison test. *P < 0.05 vs. CTR group, ^#^*P* < 0.05 vs. DEN group, ^$^*P* < 0.05 vs. SEC5 group, and ^@^*P* < 0.05 vs. SEC10 group

Hepatic levels of p-ERK, CHOP, NLRP3, IL-1β, and p-elF2α and IL-17A were significantly increased in the DEN group by 2.5-, 4.3-, 4-, 7.4-, 4.4-, and 6.7-folds, respectively in comparison to the control group (Fig. 4). Administration of SEC5, SEC10, and SEC20 significantly decreased hepatic levels of p-ERK by 26.4%, 52%, and 59%, respectively in comparison to the DEN group in a dose-dependent manner (*P* < 0.05, Fig. 4A). In addition, three doses of SEC (5, 10, and 20) elicited a significant decrease in hepatic level of CHOP by 27%, 55%, and 73%, respectively in comparison to the DEN group (*P* < 0.05, Fig. 4B). Likewise, SEC (5, 10, and 20) significantly reduced hepatic level of NLRP3 by 19.7%, 48%, and 70%, respectively compared to the DEN group (*P* < 0.05, Fig. 4C). In a dose-dependent manner, SEC (5, 10, and 20) resulted in a significant decrease in hepatic level of IL-1β by 20.5%, 46%, and 80%, respectively in comparison to the DEN group (*P* < 0.05, Fig. 4D). Furthermore, administration of SEC (10 and 20) significantly decreased hepatic level of p-elF2α by 36% and 66%, respectively compared to the DEN group (*P* < 0.05, Fig. 4E). Yet, SEC5 had no significant effect on hepatic level of p-elF2α compared to the DEN group (Fig. 4E). Administration of SEC (10 and 20) resulted in a significant decrease in hepatic level of IL-17A by 56% and 82%, respectively in comparison to the DEN group (*P* < 0.05, Fig. 4F). Yet, SEC5 had no significant effect on hepatic level of IL-17A compared to the DEN group (Fig. 4F).

### SEC attenuated DEN-induced histopathological changes in hepatic tissues

**Fig. 5:**
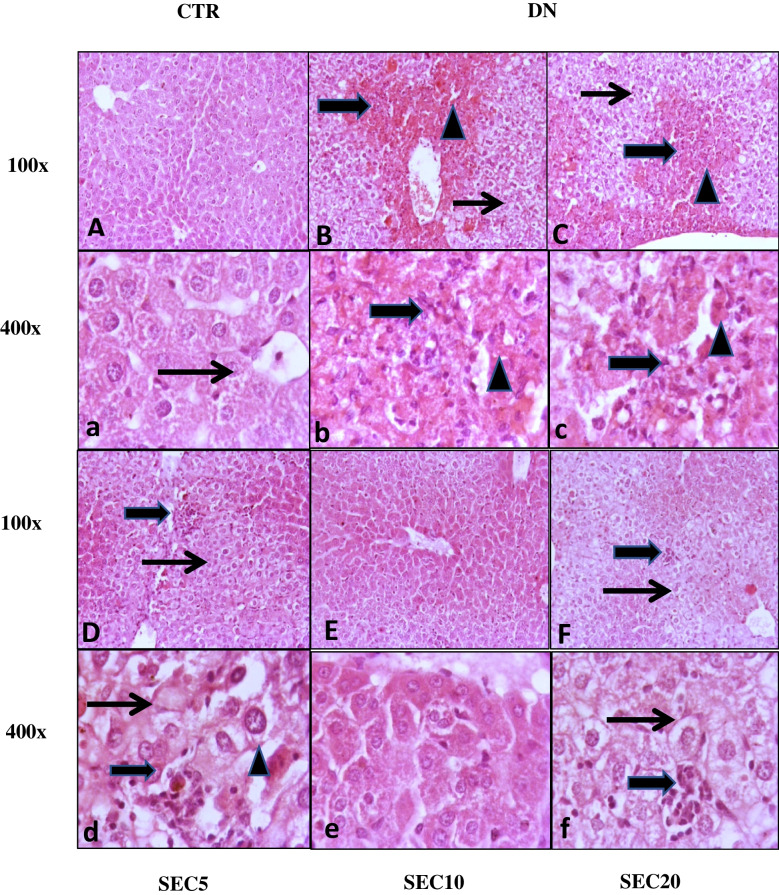
Impact of SEC on DEN-induced histopathological irregularities in hepatic sections. **A–F** Representative microscopic pictures of H&E stained hepatic sections of (**A**) Control group; **B**,** b**,** C**,** c** DEN group; **D**,** d** SEC5 group; **E**,** e** SEC10 group; and **F**,** f** SEC20 group. CTR: control, DEN: diethyl nitrosamine; SEC: secukinumab. Arrows’ indication: thin arrow—hepatocellular swelling, thick arrow—inflammation, and arrowhead—hepatocellular necrosis. Upper panel: magnification 100x. Lower panel: magnification 400x

Figure 5 represents photomicrograph of hepatic sections from different treatment groups. The Control group showed normal histological appearance of hepatic parenchyma. Hepatic sections isolated from the DEN group revealed severe perivascular zonal hepatocellular necrosis characterized by eosinophilic cytoplasm with pyknotic nuclei admixed with abundant cellular infiltrates composed of many neutrophils and few lymphocytes. Hepatic sections isolated from mice treated with SEC5 showed mild hepatocellular swelling with few hepatocyte necrosis surrounded by low numbers of inflammatory cells. Moreover, hepatic sections isolated from mice treated with SEC10 showed normal architecture with occasional hepatocellular necrosis. Treatment with SEC20 revealed mild to moderate hepatocellular swelling with few inflammatory foci.

### SEC attenuated DEN-induced changes in hepatic Caspase-1 expression

**Fig. 6:**
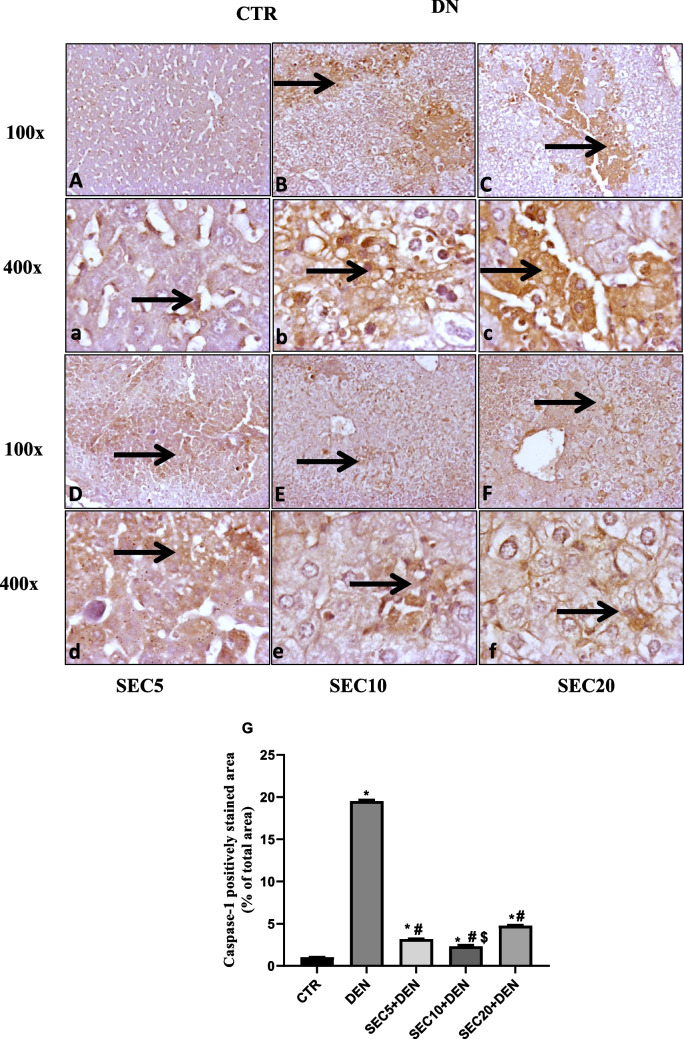
Impact of SEC on DEN-induced changes in Caspase-1 expression. **A-F** Photomicrograph of immunostained hepatic sections against Caspase-1; **G** semiquantitative analysis of hepatic Caspase-1 expression. **A** Control group; **B**,** b**,** C**,** c** DEN group; **D**,** d** SEC5 group; **E**,** e** SEC10 group; **F**,** f** SEC20 group. CTR: control, DEN: diethyl nitrosamine; SEC: Secukinumab. Arrows’ indication: thin arrow—positive immunostained hepatocytes. Upper panel: magnification 100x. Lower panel: magnification 400x. Data are expressed as means ± S.E.M. (*n* = 6). Mean values were compared via one-way ANOVA followed by post hoc Tukey’s multiple comparison test. *P < 0.05 vs. CTR group, ^#^*P* < 0.05 vs. DEN group, and ^$^*P* < 0.05 vs. SEC5 group

Immunostaining of hepatic sections against Caspase-1 showed few faint immunopositive stained hepatocytes in the control group. However, the DEN group showed high strong cytoplasmic immunopositive stained hepatocytes. However, the SEC5 group showed mild to moderate cytoplasmic expression of Caspase-1 in hepatocytes. The SEC10 group exhibited mild immunopositive stained hepatocytes. Moreover, the SEC20 group showed mild to moderate faint to high cytoplasmic expression of Caspase-1 in hepatocytes (Fig. 6). Semiquantitative analysis for Caspase-1 revealed that DEN administration significantly increased Caspase-1 expression by 19-fold as compared to the control group. Yet, administration of SEC5, SEC10, and SEC20 groups significantly decreased Caspase-1 expression by 83%, 88%, and 76%, respectively, as compared to the DEN group (Fig. 6).

### SEC attenuated DEN-induced changes in hepatic NF-κB expression

**Fig. 7:**
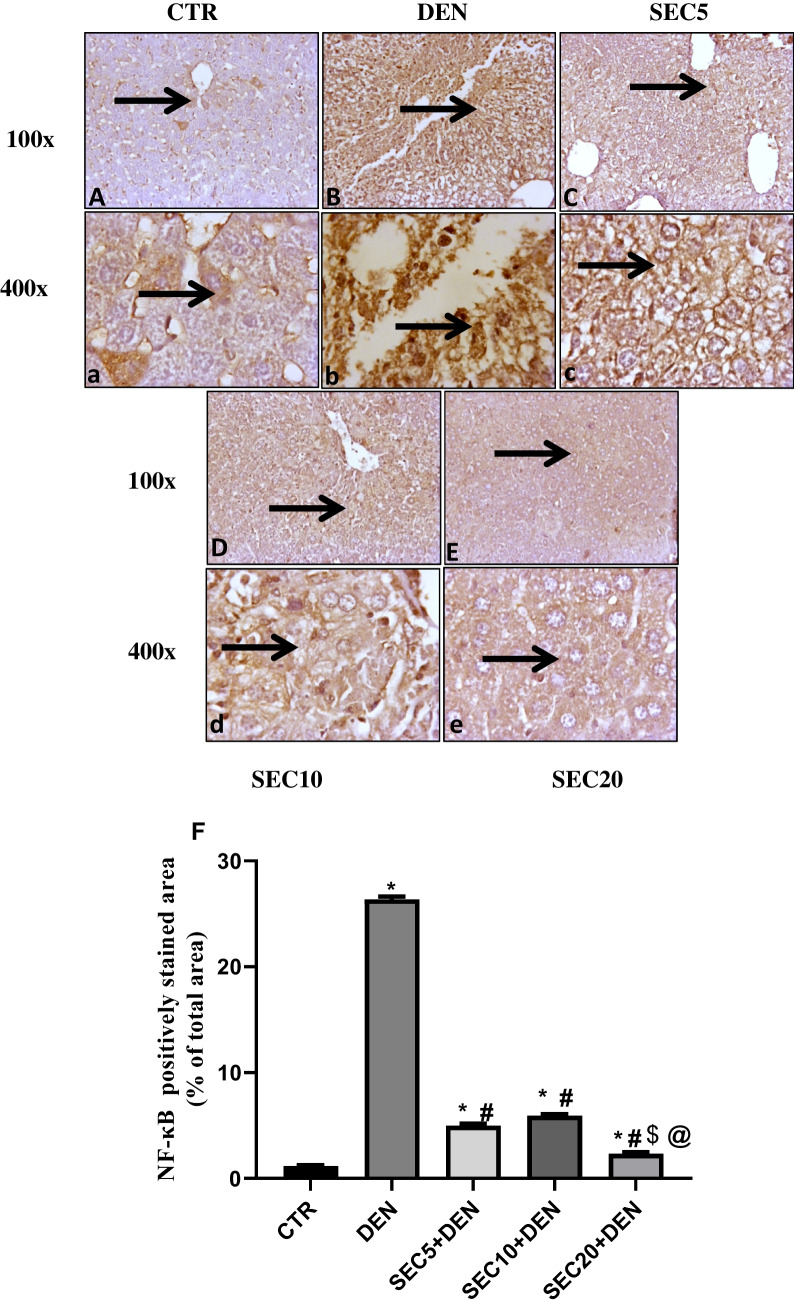
Impact of SEC on DEN-induced changes in NF-κB expression. **A–E** Photomicrograph of immunostained hepatic sections against NF-κB; **F** semiquantitative analysis of hepatic NF-κB expression. **A** Control group, **B** DEN group, **C** SEC5 group, **D** SEC10 group, and (**E**) SEC20 group. CTR: control, DEN: diethyl nitrosamine, and SEC: secukinumab. Arrows’ indication: Thin arrow—positive immunostained hepatocytes. Upper panel: magnification 100x. Lower panel: magnification 400x. Data are expressed as means ± S.E.M. (*n* = 6). Mean values were compared via one-way ANOVA followed by post hoc Tukey’s multiple comparison test. *P < 0.05, vs. CTR group, ^#^*P* < 0.05 vs. DEN group, ^$^*P* < 0.05 vs. SEC5 group, and ^@^*P* < 0.05 vs. SEC10 group

Expression of NF-κB was detected immunohistochemically on formalin-fixed hepatic sections. The control group showed few faint immunopositive stained hepatocytes. Yet, the DEN group showed diffuse strong nuclear and cytoplasmic immunopositive stained hepatocytes. However, the hepatic sections from mice treated with SEC5 showed mild to moderate expression of NF-κB in hepatocytes. In addition, the hepatic sections from mice treated with SEC10 showed diffuse moderate immunopositive stained hepatocytes. Moreover, the hepatic sections from mice treated with SEC20 showed mild expression of NF-κB in hepatocytes (Fig. 7). Semiquantitative analysis revealed that DEN administration significantly increased NF-κB expression by 22-fold as compared to the control group. Yet, administration of SEC5, SEC10, and SEC20 groups significantly decreased NF-κB expression by 81%, 77.4%, and 91%, respectively, as compared to the DEN group (Fig. 7).

## Discussion

DEN and its derivatives can be present in a variety of goods that people eat, including meat, tobacco, fried and processed meals, and alcohol. Adequate exposure to these compounds is harmful, particularly for those who are at a high risk of developing liver cancer (Shaker et al. 2022). The exact methods through which DEN causes cancer and liver damage are still unknown. According to Chowdhury et al. (2010), the main cause of DEN hepatotoxicity is metabolic activation of a-hydroxynitrosamine by microsomal cytochrome P450, which is followed by N-dealkylation to produce acetaldehyde and the reactive ethyl diazohydroxide (Chowdhury et al. 2010). The latter intermediate breaks base pairing, causes mutations, and damages DENA by alkylating DENA at several locations and forming DENA-adducts. Hepatocellular death is the outcome of such an occurrence, which is followed by compensatory proliferation, infiltration of inflammatory cells, and eventually carcinogenesis (Shaker et al. 2016).

In our study, injection of DEN induced acute liver damage as manifested by increased levels of liver enzymes, LDH as well as reduction in albumin level. These biochemical measurements were further confirmed by histopathological results which showed severe perivascular zonal hepatocellular necrosis characterized by eosinophilic cytoplasm with pyknotic nuclei admixed with abundant cellular infiltrates composed of many neutrophils and few lymphocytes. These findings agreed with earlier studies which displayed that DEN is hepatotoxic (Son et al. 2018) and can induce histopathological lesions in hepatocytes (Paula Santos et al. 2014). On the other hand, administration of secukinumab resulted in marked improvement in liver function and structure as evidenced by reduced levels of liver enzymes and improved liver architecture. This observation is consistent with an earlier study which reported that secukinumab showed hepatoprotective effect against liver fibrosis in psoriatic patients (Magdaleno-Tapial et al. 2021).

Actually, DEN metabolism produces the reactive hydroxyl radical besides the reactive metabolite, ethyl diazohydroxide. This hydroxyl radical causes oxidative DENA damage and an overabundance of lipid peroxidation aldehydes (MDA). Correspondingly, our data showed that DEN injection disturbed the oxidant/antioxidant balance as evidenced by elevated MDA content concomitant with reduced GSH level and TAC. These results came in line with previous studies which showed that DEN induced oxidative stress in hepatic tissues (Shaker et al. 2016, 2022; Zhang et al. 2021). Conversely, administration of secukinumab reversed DEN-induced alterations and restored the oxidant/antioxidant balance; secukinumab reduced MDA content and elevated TAC. These findings are in line with a former study which reported that SEC attenuated oxidative stress in a rat model of sepsis (Gao et al. 2023).

In addition to oxidative stress, inflammation is highly implicated in the pathogenesis of acute liver injury (El-Kashef and El-Sheakh 2019). IL-17A is a proinflammatory cytokine that is involved in acute liver damage via release of cytokines and chemokines, which attract neutrophils and monocytes to the site of inflammation (Zhao et al. 2022). It has been revealed that IL-17A could promote IKBα/NF-κB inflammatory signaling pathway (Sønder et al. 2011). The IKBα/NF-κB inflammatory signaling pathway activation stimulates proinflammatory cytokines including IL-1β as well as TNF-α (Su et al. 2017).

Our results showed that the inflammatory response was triggered in DEN group as verified by marked elevation in expression of NF-κB and significant increase in levels of IL-17A and IL-1β when compared to the control group. On the other hand, secukinumab markedly reduced expression of NF-κB and decreased the levels of IL-17A and IL-1β when compared to DEN group. This observation came in line with the study of Wang et al. which showed that secukinumab protected against cecal ligation and puncture (CLP)-induced sepsis through reduction in inflammatory cytokines, including IL-17A, IL-6, and TNF-α. Further findings by Wang et al. confirmed that secukinumab suppresses the IKBα/NF-κB inflammatory signaling pathway in severe sepsis by neutralizing IL-17A. This results in decreased expression of inflammation-associated cytokines and overall cytokine levels (Wang et al. 2022).

Notably, ER stress triggers the unfolded protein response (UPR), which lowers the load of unfolded proteins and improves protein folding to maintain protein homeostasis. It has been reported that UPR activation is mediated by p-ERK, a common pathway for the inflammatory response triggered by ER stress (Zhang et al. 2015). According to Cullinan and Diehl (2006), p-ERK may increase the production of proapoptotic proteins such as CHOP in advanced illnesses, which in turn would cause apoptosis (Cullinan and Diehl 2006). In this study, challenging of mice with DEN markedly induced ER stress as manifested by elevated levels of p-ERK, p-eIF2α, and CHOP. These harmful consequences may be attributed to ROS accumulation, which may cause the oxidation of resident ER proteins involving polypeptide folding machinery proteins, leading to ER stress and an intensive cycle (El-Waseif et al. 2025). On the contrary, secukinumab significantly downregulated the p-ERK/p-eIF2α/CHOP pathway when compared to the DEN group. This observation came in line with a prior study that reported that secukinumab alleviated cognitive impairment by attenuating the IL-17RA/AKT/ERK1/2 pathway (Gao et al. 2023).

NLRP3 inflammasome has a significant role in modulation of liver inflammation (Wree et al. 2018). NLRP3 activation leads to production of proinflammatory cytokines with the subsequent recruitment of neutrophils as well as other immune cells (Broderick et al. 2015). NLRP3 inflammasome may be stimulated by a variety of endogenous and external stimuli, including ROS production and ER stress (He et al. 2016). Additionally, NF-κB upregulates NLRP3 inflammasome response (An et al. 2019). Moreover, IL-17 has a significant impact in NLRP3/caspase-1 pathway modulation (Hu et al. 2025). It has been revealed that treatment with an IL-17 agonist administration could increase NLRP3 and caspase-1 expression. Also, inhibition of IL-17A can inhibit NLRP3 inflammasome activation (Zhong et al. 2022; Bai et al. 2024).

According to Kim et al. (2019), the NLRP3 inflammasome recognizes damage-associated molecular pattern (DAMP), attracts, and activates the inflammatory protease caspase-1 (Kim et al. 2019). Caspase-1 then transforms the precursor of IL-1β into its mature form, which is released to trigger inflammatory reactions (El-Waseif et al. 2025). Our data showed that there was a substantial increase in the level of NLRP3 and expression of Caspase-1 in the DEN group when compared to the control group. In contrast, secukinumab resulted in a significant reduction in NLRP3 and Caspase-1 when compared to the DEN group. This finding agreed with a previous study which showed that secukinumab suppressed IL-17-mediated pyroptosis in sunitinib-induced renal toxicity (Elrashidy et al. 2025).

**Fig. 8:**
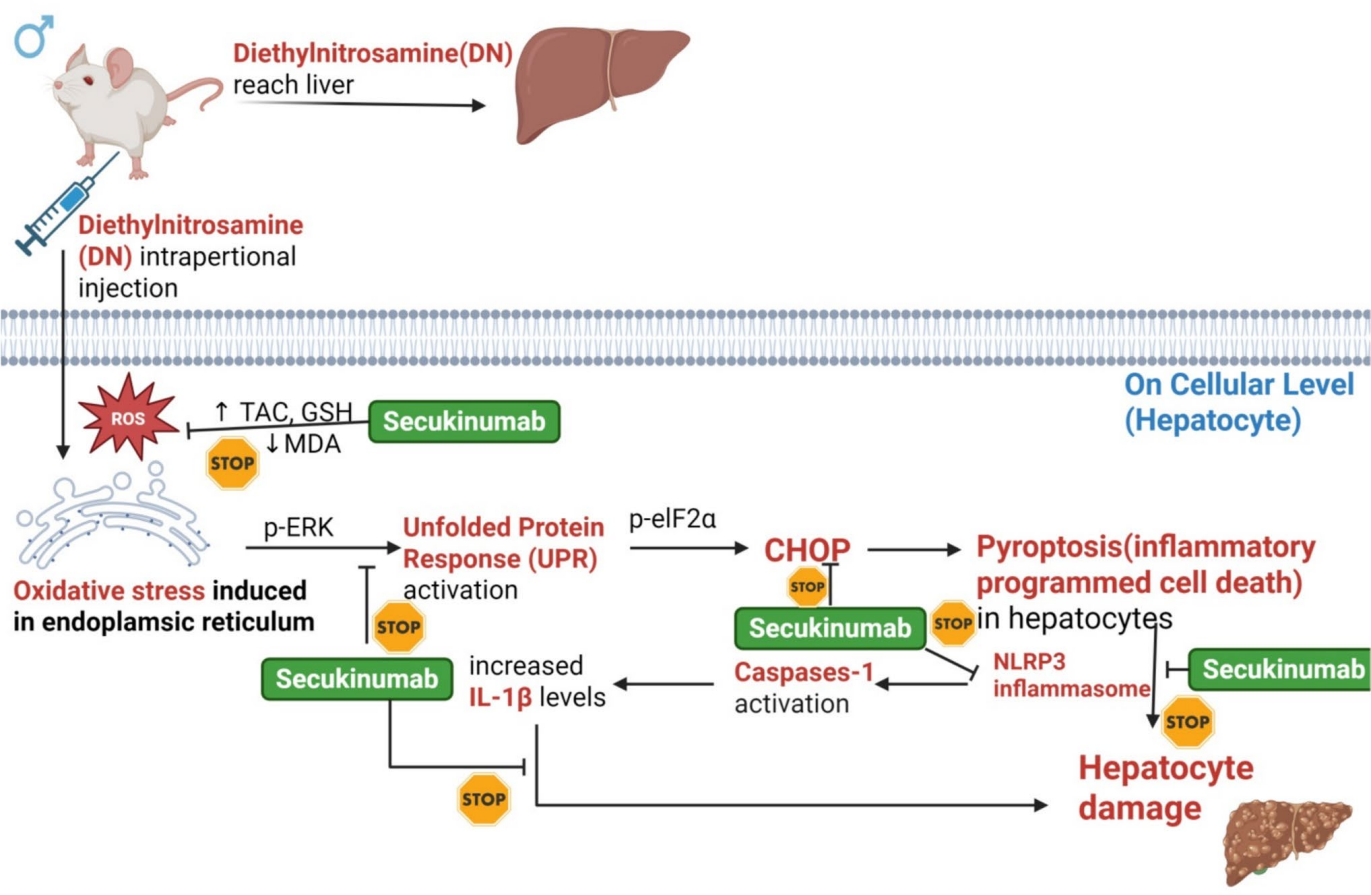
Proposed mechanism of hepatoprotective effect of Secukinumab against diethyl nitrosamine-induced acute liver injury. CHOP: C/EBP homologous protein, GSH: reduced glutathione, IL-1β: interleukin-1β; MDA: malondialdehyde; NF-κB: nuclear factor-κB, NLRP3: NLR family, pyrin domain containing protein 3; p-elF2α: phosphoeukaryotic translation initiation factor 2 subunit; p-ERK: phosphorylated extracellular signal-regulated kinase; TAC: total antioxidant

Collectively, the findings of the current study obviously demonstrated the hepatoprotective benefits of secukinumab against diethylnitrosamine-induced acute liver injury in mice, potentially by reducing ROS and restoring the antioxidant/oxidant balance. Furthermore, secukinumab greatly reduced ER stress by inhibiting the p-ERK/p-eIF2α/Chop pathway and the IL-17A/NLRP3/caspase-1/IL-1β pyroptotic pathway, both of which preserve liver integrity. Further investigations are required to prove its therapeutic effect in clinical use. The proposed mechanism of the hepatoprotective effect of secukinumab is illustrated in Fig. 8.

### Limitation of the study

This study focused on the preventive role of SEC rather than the therapeutic role. Thus, the therapeutic impact of SEC against DEN-induced hepatotoxicity needs to be considered in our future studies.

## Data Availability

All source data for this work (or generated in this study) are available upon reasonable request.
